# Identifying Clinical and MRI Characteristics Associated with Quality of Life in Patients with Anterior Cruciate Ligament Injury: Prognostic Factors for Long-Term

**DOI:** 10.3390/ijerph182312845

**Published:** 2021-12-06

**Authors:** Eleuterio A. Sánchez Romero, Tifanny Lim, José Luis Alonso Pérez, Matteo Castaldo, Pedro Martínez Lozano, Jorge Hugo Villafañe

**Affiliations:** 1 Musculoskeletal Pain and Motor Control Research Group, Faculty of Sport Sciences, Universidad Europea de Madrid, Villaviciosa de Odón, 28670 Madrid, Spain; tifanny94@live.fr (T.L.); joseluis.alonso@universidadeuropea.es (J.L.A.P.); pedro.martinez@universidadeuropea.es (P.M.L.); 2 Department of Physiotherapy, Faculty of Sport Sciences, Universidad Europea de Madrid, Villaviciosa de Odón, 28670 Madrid, Spain; 3 Musculoskeletal Pain and Motor Control Research Group, Faculty of Health Sciences, Universidad Europea de Canarias, La Orotava, Tenerife, 38300 Canary Islands, Spain; 4 Department of Physiotherapy, Faculty of Health Sciences, Universidad Europea de Canarias, La Orotava, Tenerife, 38300 Canary Islands, Spain; 5 Onelifecenter, Multidisciplinary Pain Treatment Center, 28925 Madrid, Spain; 6 Center for Neuroplasticity and Pain (CNAP), Center for Sensory-Motor Interaction (SMI), Department of Health Science and Technology, Faculty of Medicine, Aalborg University, 9220 Aalborg, Denmark; matteo.castaldo@poliambulatoriofisiocenter.com; 7 Sport Physiotherapy, University of Siena, 53100 Siena, Italy; 8 Instituto di Ricovero e Cura a Carettere Scientifico (IRCCS) Fondazione Don Carlo Gnocchi, 20141 Milan, Italy

**Keywords:** knee injuries, anterior cruciate repair, bone contusion

## Abstract

Background: Associated lesions in the diagnostic MRI may be related to worse long-term subjective outcomes. There is a lack of conclusive information about the long-term outcomes of associated injuries in anterior cruciate ligament (ACL) tears. The purpose of this study is to assess the long-term effects of associated injuries in ACL tears measured by means of a quality of life (QOL) assessment. Methods: A retrospective cohort study of 225 consecutive patients admitted for physical therapy with ACL injury (42 ± 12 years, 28.2% female) were conducted. All demographic and clinical variables were used to measure a QOL. Univariate and multivariable analyses were completed. Results: The mean follow-up period was 8.4 ± 2.6 years. In univariate analysis, male gender, and sports as the cause of the ACL lesion were factors significantly associated with improved International Knee Documentation Committee (IKDC) scores at the end of follow-up (all *p* < 0.002). In multivariable analysis, the occurrence of bone contusion was positively associated with injury (OR = 2.12) and negatively associated with sports injury (OR = 0.44) and medial collateral ligament (MCL) injury (OR = 0.48). Conclusions: After ACL injury, male gender and sports injury were associated with better clinical outcomes.

## 1. Introduction

Anterior cruciate ligament tears are common and affect young individuals who participate in jumping and pivoting sports [1]. There is ample evidence that on long-term follow-up, these lesions are associated with the development of knee osteoarthritis (OA), leading to pain and functional impairment in impairment in young and middle-aged adults: the young patient with an old knee [2,3]. Injured patients report knee instability, which restricts activity and affects the quality of life (QOL) [4,5,6]. Accuracy in the diagnosis of ACL rupture is obtained by anamnesis and clinical examination of the patient, in addition to complementary imaging studies [6]. In addition, there are common injuries associated with ACL tears, such as meniscal, cartilage, and other ligament damage in the knee joint complex, and it is especially important that these injuries are also properly diagnosed [7]. However, symptomatic OA in these young patients remains a profound and largely unsolved treatment challenge [8]. The treatment of ACL injuries is still highly controversial, due to limited conclusive data based on comparative studies on the factors affecting the long-term outcome of these injuries, although recent prospective studies have defined short-term factors predicting rapid recovery [9]. While there are studies that recommend earlier surgical reconstruction after ACL injury in order to prevent further meniscal damage and to decrease the risk of degenerative arthritis, other authors recommend a rehabilitation program and optional ACL reconstruction for those cases in which the patient really requires it [10,11]. On the other hand, a few studies demonstrated that good functional results could be obtained by conservative measures in the majority of subjects with unilateral unreconstructed ACL injury [12]. Although there is consensus that the rehabilitation program should be based on gaining strength, range of motion, proprioception, development of functionality according to the patient’s requirements, and sport-specific training (if applicable) in addition to psychological testing; motor control training has been shown to be useful in reducing the risk of a second ACL injury and early development of OA [6,13,14]. In this same sense, it has been observed that the performance of neuromuscular exercises improves the timing of activation of the knee stabilizing muscles, thus potentially reducing the risk of ACL injury [15,16,17,18]. It should also be pointed out that the joint tissues do not appear to be the only cause of pain and dysfunction in the patient, but that the periarticular muscles are also involved in the clinical presentation of many patients [19,20]. For the evaluation of the results after treatment, it is essential to evaluate the QOL, in addition to functionality by means of the clinical history, physical examination, validated questionnaires, and complementary tests [6,8].

The long-term effects of associated lesions on the ACL injury are still debated [21]. Early identification of long-term prognostic factors may allow for a more appropriate treatment plan and better information for the patient as it could result in advice on lifestyle change [22]. In this sense, it appears that lifestyle modifications and fear of re-injury influence patients’ quality of life even 20 years after injury, and that it is patients with a preference for competitive sport who are at the greatest risk of worse QOL [23]. Long-term studies are difficult to perform for different reasons such as the elevated cost of their development or the high dropout rate of study subjects, in addition to the ethical reasons for assessing certain aspects of the injury prospectively. In the last two decades, information and communication technologies have been introduced in clinical practice, allowing for the prospective recording of data and appropriate identification of subjects potentially contributing to post-hoc designed studies on prognosis. Therefore, the aim of the present study was to retrospectively evaluate the long-term effects of injuries associated with anterior cruciate ligament (ACL) ruptures, as measured by IKDC score (QOL assessment).

## 2. Materials and Methods

### 2.1. Study Design

We conducted a retrospective cross-sectional study on male and female patients with an MRI diagnosis of ACL injury on a study performed between September 2010 and July 2015. Procedures were conducted following the Strengthening the Reporting of Observational Studies in Epidemiology (STROBE) statement and checklist [24]. The study protocol was approved by the Ethical Committee of the Universidad Rey Juan Carlos, Madrid, Spain (reference number 2008202016220). Informed consent was obtained from all participants, and all procedures were conducted according to the Declaration of Helsinki.

### 2.2. Participants

All participants were assessed via a comprehensive clinical anamnesis and objective physical examination performed by two expert physical therapists in a rehabilitative clinic between September 2020 and November 2020. To be included in the study, the patients needed to present an ACL injury without signs of meniscal injury diagnosed by MRI and be of adult age (over 18 years). Exclusion criteria included: previous ipsilateral knee surgery, central or peripheral neurologic signs, systemic illness (tumor and rheumatologic diseases), recent unrelated lower limb pathology or trauma, and limiting psychiatric pathology.

### 2.3. Clinical Measurements

For the analysis of the knee MRI data, the following information was collected: date of MRI, left/right knee, detail of ACL/posterior cruciate ligament (PCL)/medial and/or lateral meniscus/collateral ligaments injury, and presence and location of bone contusions, other associated knee injuries (cartilage, tendon, posterolateral corner injury, fractures, inflammatory changes), as well as any other associated injury findings. Information was also collected from electronic medical records on patient demographics, treatment options (surgical/conservative), details of surgical approach and possible complications, age, height, weight, sex, work, physical activity, mechanism of injury, and possible association with sports [6,25,26]. Functional outcome score (The International Knee Documentation Committee (IKDC) score) was obtained and considered the main clinical outcome variable. The IKDC evaluation form is a validated instrument to record the clinical state of ACL reconstructed patients at one time [27,28]. The IKDC scores form was used to measure QOL in our statistical analysis. This form, with Likert-type scale questions, transforms the raw points to a scale from 0 to 100, with 100 being the maximum score for functional independence and absence of pain. All the clinical information, general patient information, type of treatment received, post-treatment events, and MRI evaluation were evaluated as prognostic factors for the clinical outcome. Secondarily, factors predicting the presence of bone contusion on MRI were evaluated in a different analysis.

### 2.4. Statistical Analysis

Data were analyzed using SPSS version 25.0 (SPSS IBM Inc, Chicago, IL, USA) was used to record demographic, clinical, and radiological variables. The descriptive statistics were calculated for IKDC, age, height, and weight. All variables in the univariate analysis with a *p* < 0.10 were entered into a logistic regression model. To identify factors predicting outcome (age, height, weight, sex, job, physical activity, injury mechanism, and whether it was sports-associated), two separate binary logistic regression models were constructed to differentiate between bone contusion (yes/no) outcomes. A multivariable analysis was carried out in which all potential predictors were included. The potential predictors were identified using univariate analysis and expert opinion. The performance of the models was assessed using mean squared errors and R^2^ values.

## 3. Results

Two hundred and twenty-five consecutive patients with ACL injury cases were screened for eligibility criteria. One hundred and ninety-five patients (43 ± 10.3 years, 30% female) satisfied all eligibility criteria. The mean follow-up period was 8.4 ± 2.6 years. The remaining patients that met the inclusion criteria were not followed-up because they did not respond to any of the three attempts of contact, had changed telephone numbers, were reported deceased, or did not wish to partake in the study, Table 1.

In univariate analysis, male gender, and sports as the cause of the ACL lesion were factors significantly associated with better IKDC scores at the end of follow-up (all *p* < 0.002) (Table 2).

The cut-off points were approximately 78.5 IKDC for the bone contusion (81.3% sensitivity; 66.7% specificity) and 77.5 IKDC for the sport injury (85.0% sensitivity; 45.2% specificity).

Multivariable logistic regression could not demonstrate a combination of factors that was significantly associated with the IKDC score. In multivariable analysis, the occurrence of bone contusion was positively associated to associated injury (OR = 2.12; *p* = 0.02), sports injury (OR = 0.44; *p* = 0.04) and MCL injury (OR = 0.48; *p* = 0.04). The final regression model explained 74.3% (R^2^ = 0.08; *p* = 0.002) of the variability for the occurrence of bone contusion (Table 3).

## 4. Discussion

The present study evaluated the long-term findings detailed as an ACL injury-associated IKDC score (QOL) in a large sample of subjects in order to establish prognostic factors so that patients can be informed of the long-term consequences of an ACL injury. Clinical history, age, height, weight, sex, work, laterality, physical activity, mechanism of injury, association with sport, as well as MRI findings in a single radiology department of a single orthopedic center, were evaluated as possible prognostic factors predicting outcome. Finally, male gender and sports as the cause of the ACL lesion were factors significantly associated to better IKDC scores (QOL) at the end of follow-up.

We found bone contusions were positively associated with other injuries. Bone contusions are the consequence of an impact force on the bone, and when associated with an ACL injury, they may proceed to a complete resolution. However, a certain proportion of bone contusions can persist a year following the injury, and can be associated with early signs of osteoarthritis [29]. They have long been suspected as a risk predictor for worse outcomes following ACL injury [30], although to our knowledge, no data on this association has been published. Qiu et al. [31] performed a retrospective study by analyzing 93 MRI images of patients who had suffered an acute non-contact ACL injury to identify bony contusions of the knee by MRI scans for each anatomic femoral and tibial joint site. They found a prevalence of bone contusions of 78.49% at the lateral femoral condyle, 88.17% at the lateral tibial plateau, 49.46% at the medial femoral condyle, and 69.89% at the medial tibial plateau; determining that the location patterns and severity of bone contusions in patients indicated that tibial internal rotation, valgus, and anterior and lateral tibial translation were the main mechanisms of non-contact ACL injury. Due to the possibility to develop early knee OA, searching for the presence of bone contusion may be interesting to evaluate the long-term effects of the ACL injury [29].

It is now accepted that ACL injuries are associated with the development of knee OA [32], although a significant number of patients may be asymptomatic and require treatment more than 30 years after ACL injury [33]. Knee injury recurrence and the development of post-traumatic knee OA are potential risks in patients who have suffered ACL injury [13,14,26]. It appears that a change in lifestyle, modifying sport and/or physical activity to avoid performing sports/movements of pivoting and shear [34,35], and learning through motor control training has been shown to be useful in reducing the risk of a second ACL injury and early development of OA [6,13,14,36]. In addition, there is a higher risk of knee OA in patients with higher BMI and in women, being physically inactive and having muscle weakness of the knee muscles [37,38]. Likewise, an association between suffering an ACL lesion in youth and the subsequent development of obesity has been demonstrated [39].

It should be noted that it is important to maintain or initiate physical activity in the patient who has sustained an ACL injury in order to achieve and enjoy adequate levels of QOL [23]. Therefore, educational strategies should be established to address all these modifiable risk factors.

Conservative treatment by muscle stabilization of the knee is effective and can help to reduce or avoid the complications of surgical treatment [40]. Since it is known that neuromuscular alterations are a direct consequence of knee injuries and are associated with an increased risk of early post-traumatic OA and that neuromuscular alterations continue for years after ACL injury accompanied by neural alterations and changes in the control and synchronization of muscle strength, knee motor control training is strongly recommended, along with a battery of strength and jumping tests, quality of motion examination and quality of motion work, and psychological tests to guide progression from one phase of rehabilitation to the next [13,41,42].

Forbel et al. [11] investigated ACL treatment interventions by dividing 121 young, active adults with an acute ACL injury into two groups of structured-based rehabilitation of a four-level program of exercises and goals aimed at gaining a range of motion, muscle function, and functional performance + ACL reconstruction surgery, or the same structured-based rehabilitation protocol and the option of later ACL reconstruction if needed. They found and concluded that the second group of structure-based rehabilitation + elective surgery substantially reduced the need for surgical intervention, with rehabilitation being effective for many patients as the sole treatment in that group.

A physiotherapy program should be supervised and is associated with acceptable results also in specific cases of low-requirement patients [43]. Kise et al. [44] concluded by analyzing 140 middle-aged patients with MRI-verified degenerative medial meniscus tears that treatment interventions of supervised exercise therapy for 12 weeks was superior to arthroscopic partial meniscectomy for knee function.

We have found an association between male gender and sport-related ACL injury and better clinical outcomes. This may reflect the overall effect of general health or physical performance on long-term outcomes. Psychological factors might also partly explain these results. The significance of the discrepancies shown in this study suggests that treatment modality (surgical vs. conservative) is more effective when patients are informed about the long-term prognosis.

### Limitations

Some possible limitations of the present study could have been reduced by detailing the type of sport and type of surgery (in cases where operative treatment was chosen) among the patients of the present study. In addition, it should be noted that no information was collected on the degree of injury or rupture as measured by MRI.

Although it is true that the inherent bias on the quality of the information that exists in any retrospective study design has been minimized thanks to the use of a very complete and reliable data source, it should be noted that the socioeconomic level to which the patients included in the study belonged was not taken into account, nor was the type of diet they maintained [45,46,47].

## 5. Conclusions

Better clinical outcomes were associated with male sex and sports injury at a follow-up of more than eight years. Well-defined and adequately powered prospective cohort studies are needed to better understand the role of preoperative and postoperative variables and treatment modalities, which will allow a better definition of prognostic factors in ACL injuries. This knowledge is useful for treatment choice and patient counseling.

## Figures and Tables

**Table 1:** Baseline demographics for both groups.

Variable	Surgery (*n* = 195)
Age (years)	43.3 ± 10.3
Gender, (*n* (%))	
Male	156 (80%)
Female	39 (20%)
Height (m)	175.6 ± 7.6
Weight (kg)	75.4 ± 13.5
IKDC (total)	87.1 ± 11.6
Follow up (years)	8.4 ± 2.6
ACL injury, (*n* (%))	
Partial tears	39 (20%)
Total tears	156 (80%)
PCL injury, (*n* (%))	
Yes	3 (1.5%)
No	192 (98.5%)
MCL injury, (*n* (%))	
Yes	58 (29.7%)
No	137 (70.3%)
ECL injury, (*n* (%))	
Yes	11 (5.6%)
No	184 (94.4%)
Bone contusion, (*n* (%))	
Yes	121 (62.1%)
No	74 (37.9%)
Sport injury, (*n* (%))	
Yes	146 (74.9%)
No	49 (25.1%)
Sport associated, (*n* (%))	
Yes	171 (87.7%)
No	24 (12.3%)

Abbreviations: ACL: Anterior cruciate ligament; PCL: Posterior cruciate ligament; MCL: Medial collateral ligament, ECL: External collateral ligament.

**Table 2:** Univariate analysis of associations with total IKDC score.

Variable	N	IKDC Score	Mean Difference	95% CI	*p*
*Gender*					
Male	156	88.5 ± 13.2	6.2#	2.1; 10.2	0.002
Female	39	82.3 ± 15.1			
*ACL*					
Acute, partial tears	39	87.0 ± 11.3	−0.2	−4.8; 4.3	0.8
Acute, complete tears	156	87.2 ± 13.7			
*Bone contusion*					
Yes	121	89.8 ± 13.1	−4.0	−8.3; −2.7	0.2
No	74	93.7 ± 14.2			
*Sports injury*					
Yes	171	88.1 ± 14.7	11.8#	7.3; 16.4	0.001
No	24	76.2 ± 15.8			
*Treatment of ACL lesion*					
Surgical	137	82.7 ± 14.6	−2.3	−6.3; 1.8	0.4
Conservative	58	85.0 ± 13.7			
*Side*					
Right	107	86.4 ± 14.2	−1.1	−3.6; 3.5	0.9
Left	88	87.6 ± 13.9			
*PCL*					
Injury	3	76.8 ± 9.3	−8.6	−25.2; 7.6	0.3
No injury	192	85.4 ± 14.5			
*MCL*					
Injury	61	85.9 ± 15.5	−0.9	−4.8; 3.1	0.7
No injury	134	86.8 ± 13.9			
*ECL*					
Injury	11	85.2 ± 17.1	−1.4	−9.1; 6.6	0.8
No injury	184	86.6 ± 13.2			

Abbreviations: ACL: Anterior cruciate ligament; PCL: Posterior cruciate ligament; MCL: Medial collateral ligament, ECL: External collateral ligament.

**Table 3:** Independent predictors of consequential bone contusion on the logistic regression model.

					95% Confidence Interval for Bone Contusion Relative Risk	
Factors	B	Wald	*p*-Value *	Bone Contusion Odds Ratio	Lower Bound	Upper Bound
Intercept	−0.39	1.02	0.31	-	-	-
Associated injury (Ref: no)	−0.75	5.46	0.02 *	2.12	1.07	3.92
Sports injury (Ref: no)	−0.82	0.41	0.04 *	0.44	0.22	0.97
*MCL* injury (Ref: no)	−0.74	4.31	0.04 *	0.48	0.23	0.95

Abbreviations: MCL: Medial collateral ligament. * Data are expressed as means ± standard deviations (SD).

## Data Availability

The data presented in this study are available on request from the corresponding authors.
